# Evaluation of a curved intramedullary button vs. traditional flat button for proximal biceps tenodesis: a biomechanical study

**DOI:** 10.1016/j.jseint.2025.03.020

**Published:** 2025-04-16

**Authors:** Igor J. Shirinskiy, Arno A. Macken, Pieter Caekebeke, Derek F.P. van Deurzen, Gabriëlle J.M. Tuijthof, Tjarco D.W. Alta, Ronald L.A.W. Bleys, Rob P.A. Janssen, Michel P.J. van den Bekerom

**Affiliations:** a Faculty of Behavioral and Movement Sciences, Department of Human Movement Sciences, Vrije Universiteit Amsterdam, Amsterdam Movement Sciences, Amsterdam, The Netherlands; b Shoulder and Elbow Unit, Department of Orthopedic Surgery, OLVG, Amsterdam, The Netherlands; c Alps Surgery Institute, Clinique Générale d'Annecy, Annecy, France; d Department of Orthopaedics and Sports Medicine, Erasmus MC, Rotterdam, The Netherlands; e Department of Orthopaedics and Traumatology, Ziekenhuis Oost-Limburg, Genk, Belgium; f Faculty of Rehabilitation Sciences, University of Hasselt, Hasselt, Belgium; g Amsterdam Shoulder and Elbow Centre of Expertise (ASECE), OLVG, Amsterdam, The Netherlands; h Department of Biomechanical Engineering, University of Twente, Enschede, The Netherlands; i Department of Orthopaedics and Traumatology, Spaarne Gasthuis, Haarlem/Hoofddorp, The Netherlands; j Department of Anatomy, University Medical Center Utrecht, Utrecht, The Netherlands; k Department of Orthopaedic Surgery & Trauma, Máxima Medical Center, Eindhoven, The Netherlands; l Department of Biomedical Engineering, Orthopaedic Biomechanics, Eindhoven University of Technology, Eindhoven, The Netherlands; m Department of Paramedical Sciences, Health Innovations & Technology, Fontys University of Applied Sciences, Eindhoven, The Netherlands

**Keywords:** Proximal, Fixation, Intramedullary, Button, Repair, Tendon, Biceps

## Abstract

**Background:**

Although variations in fixation techniques for proximal biceps tenodesis have been evaluated by different biomechanical studies, the optimal fixation technique remains unclear. To further advance the biceps tenodesis technique using unicortical button, a novel curved button was developed. This study aimed to compare the time-zero biomechanical performance of a curved intramedullary button with that of a standard flat button in proximal biceps tenodesis.

**Methods:**

A total of 16 cadaveric fresh-frozen shoulders were randomly allocated to undergo proximal biceps tenodesis using either a standard flat button or the new curved button (developed by Materialise, Leuven, Belgium). Following the tenodesis, the biceps tendon was subjected to a cyclic load, ranging from 5 to 100N, at a frequency of 2.5 Hz for a total of 1000 cycles. After this, the tendon was loaded to failure. During testing, displacement after cyclic loading, ultimate failure load, stiffness, and modes of failure were evaluated.

**Results:**

All 16 specimens were included in the data analysis. No failure occurred during cyclic testing. After cyclic testing, the median displacement was 10.4 mm (6.1-16.1) for curved button and 11.9 mm (6.0-43.9) for flat button (*P* = .534). The mean ultimate load to failure for the curved button was 239.4 ± 35.1 N, and 227.0 ± 42.0 N for the flat button (*P* = .721). The mean stiffness was 70.0 ± 10.3 N/mm for the curved button and 61.3 ± 17.3 N/mm for the flat button (*P* = .242).

**Conclusions:**

In this time-zero in-vitro study, the curved and flat buttons exhibited similar biomechanical properties in terms of displacement, load to failure, and stiffness. Considering these results and the theoretical advantages of the curved button, this technique could be a new alternative for the treatment of proximal long-head biceps tendon pathology.

The purpose of biceps tenodesis is to treat biceps tendon pathologies, including tenosynovitis, tears, instability, or superior labrum anterior-posterior lesions, by surgically reattaching the biceps tendon to the humerus.^6^ This procedure aims to relieve pain and improve function in the affected shoulder. Numerous fixation techniques of the proximal bicep tenodesis have been described. Depending on the suprapectoral or subpectoral location of the proximal biceps tenodesis, the long-head biceps tendon can be secured by various techniques, such as interference screw, suture anchor, cortical button, or soft tissue tenodesis.

Although variations in proximal biceps tenodesis fixation techniques have been evaluated by different biomechanical studies, the optimal fixation technique remains unclear. Data from systematic reviews suggest that the interference screw is the strongest fixation technique.^1^^,^^7^ However, the interference screw has some limitations. Fixation with an interference screw is a procedure that carries the risk of stripping the cortical bone or cutting the tendon with the screw, which can cause failure of tenodesis.^2^^,^^13^ A biomechanical study by Buchholz et al, that compared tenodesis using interference screw and intramedullary cortical button, reported a 30% failure rate for the tenodesis with interference screw.^4^ Furthermore, placement of the cortical screw requires a larger tunnel than the placement of cortical button.^2^ Therefore, cortical button has a lower fracture risk than cortical screw and withstands a larger torsional force during biomechanical testing.^12^ Additionally, a recent biomechanical study showed that a cortical button was stronger than interference screw.^8^ Based on this, the cortical button delivers equivalent strength, is a simpler technique, and has a decreased fracture risk.

To further advance the biceps tenodesis technique with the unicortical button, a novel curved button was developed.^5^ This button is different from the flat unicortical buttons that are currently in use, which depend on an onlay technique involving the placement of the biceps tendon on the surface of humeral bone. Due to its curved shape, this new button for unicortical fixation makes it possible to achieve an inlay tendon fixation, which places the tendon inside the cortical tunnel without the need for bicortical drilling (Fig. 1). This avoids the increased risk of neurologic injury that is present during bicortical drilling, as previously described by Sethi et al.^17^ While achieving a partial intramedullary insertion, this intracortical placement aims to potentially combine cortical and in-tunnel healing. This partial inlay technique may offer a biomechanical advantage, particularly regarding initial fixation strength during the early healing phase. This is supported by a study suggesting enhanced fixation strength with intracortical placement.^10^ Additionally, this inlay technique could maintain tendon proximity to the cortical tunnel even after the potential initial tendon slippage during early mobilization, which may result in better tendon-bone healing (Fig. 1*A* vs. *C*).^2^^,^^18^ To date, there have been no studies performed on proximal biceps tenodesis with a curved button. Therefore, the purpose of this study was to compare the time-zero biomechanical performance of a novel curved intramedullary button to a standard flat button in proximal biceps tenodesis. Based on previous studies, the hypothesis was that both fixation techniques would demonstrate comparable fixation strength.^1^^,^^7^^,^^8^

**Figure 1:**
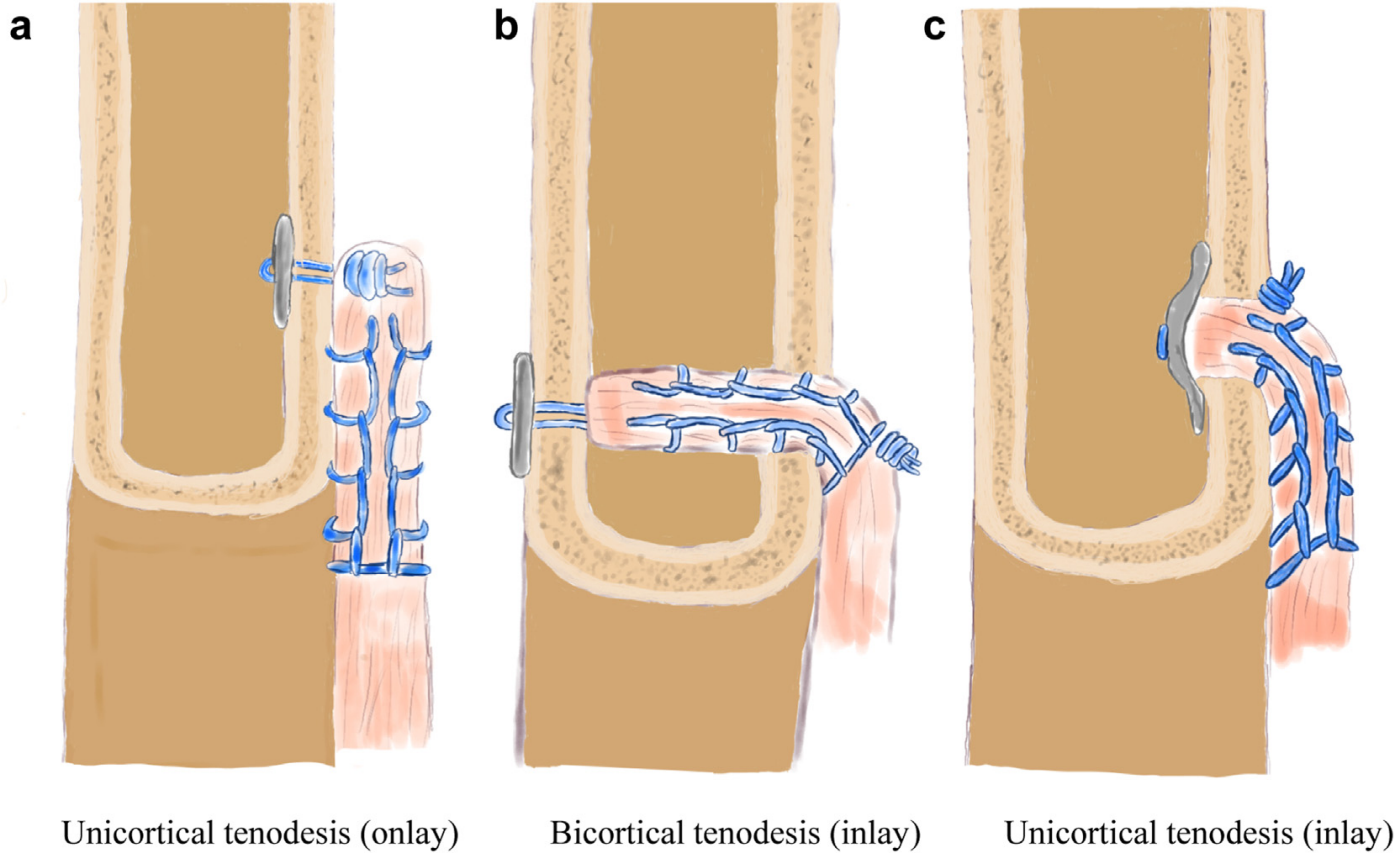
(**a**-**c**) Schematic overview of different button tenodesis fixation techniques.

## Material and methods

### Specimen preparation

Sixteen fresh-frozen human shoulders were randomly allocated between two groups. These specimens were derived from bodies that had entered the department of anatomy of University Medical Center Utrecht through a donation program. From these persons, written informed consent was obtained during life that allowed the use of their entire bodies for educational and research purposes. In the first group, biceps tenodesis was performed with the standard flat unicortical button typically used for biceps tenodesis, and in the second group, biceps tenodesis was performed with the newly developed curved button.

### New button design

**Figure 2:**
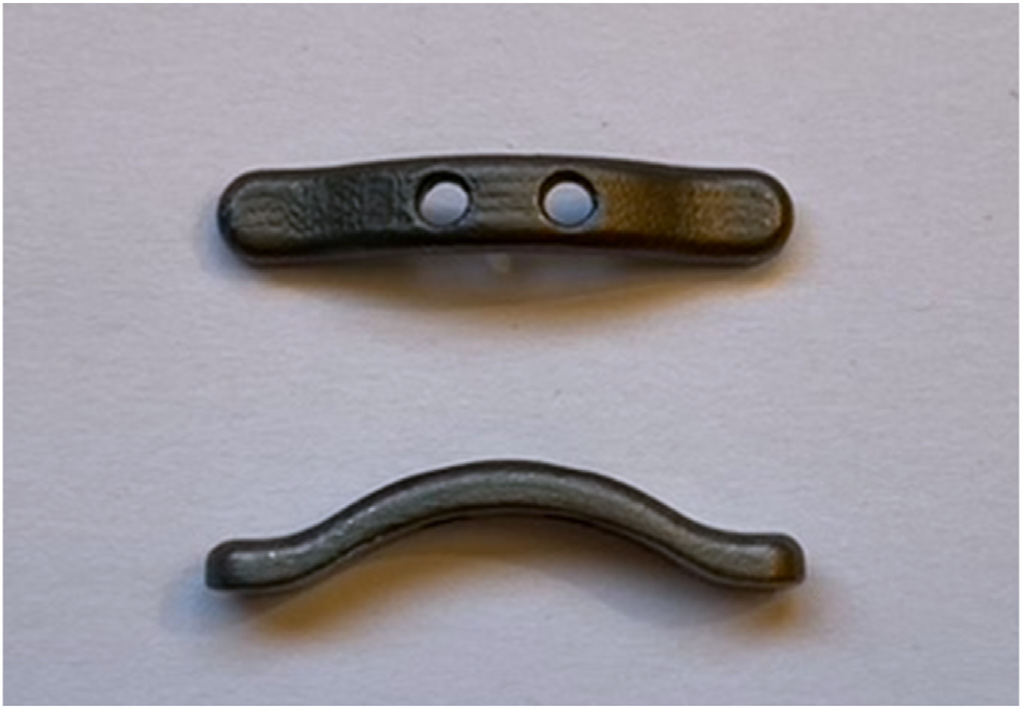
Curved button.

The new curved button (Materialise, Leuven, Belgium) (Fig. 2) is printed in titanium and was designed using 3D software (Autodesk Fusion 360; Autodesk, San Francisco, CA, USA). This button was evaluated on 12 radius specimens to establish its size as it was initially intended for the tenodesis of the distal biceps tendon. The design features a curved shape to allow the tendon to be pulled into the hole in the cortical bone with a maximum depth of 3 mm plus the thickness of the proximal cortex. The button has a width of 4 mm, thickness of 2 mm, and a length of 23 mm.

### Surgical technique and biomechanical testing

First, the long-head biceps tendon was detached from its attachment point in the labrum through a mini-open incision. Subpectoral biceps tenodesis was performed through a three-to four-centimeter incision at the anteromedial aspect of the proximal humerus, centered over the inferior border of the pectoralis major tendon. After locating the long-head biceps tendon, the tendon is pulled out of the incision and transected two to three centimeters proximally to the musculotendinous junction. A nonabsorbable suture (ULTRABRAID (#2); Smith and Nephew, Watford, England, UK) was passed in a Krackow stitch fashion through the tendon with its ends emerging at the tendon stump. The suture was passed through the button.

**Figure 3:**
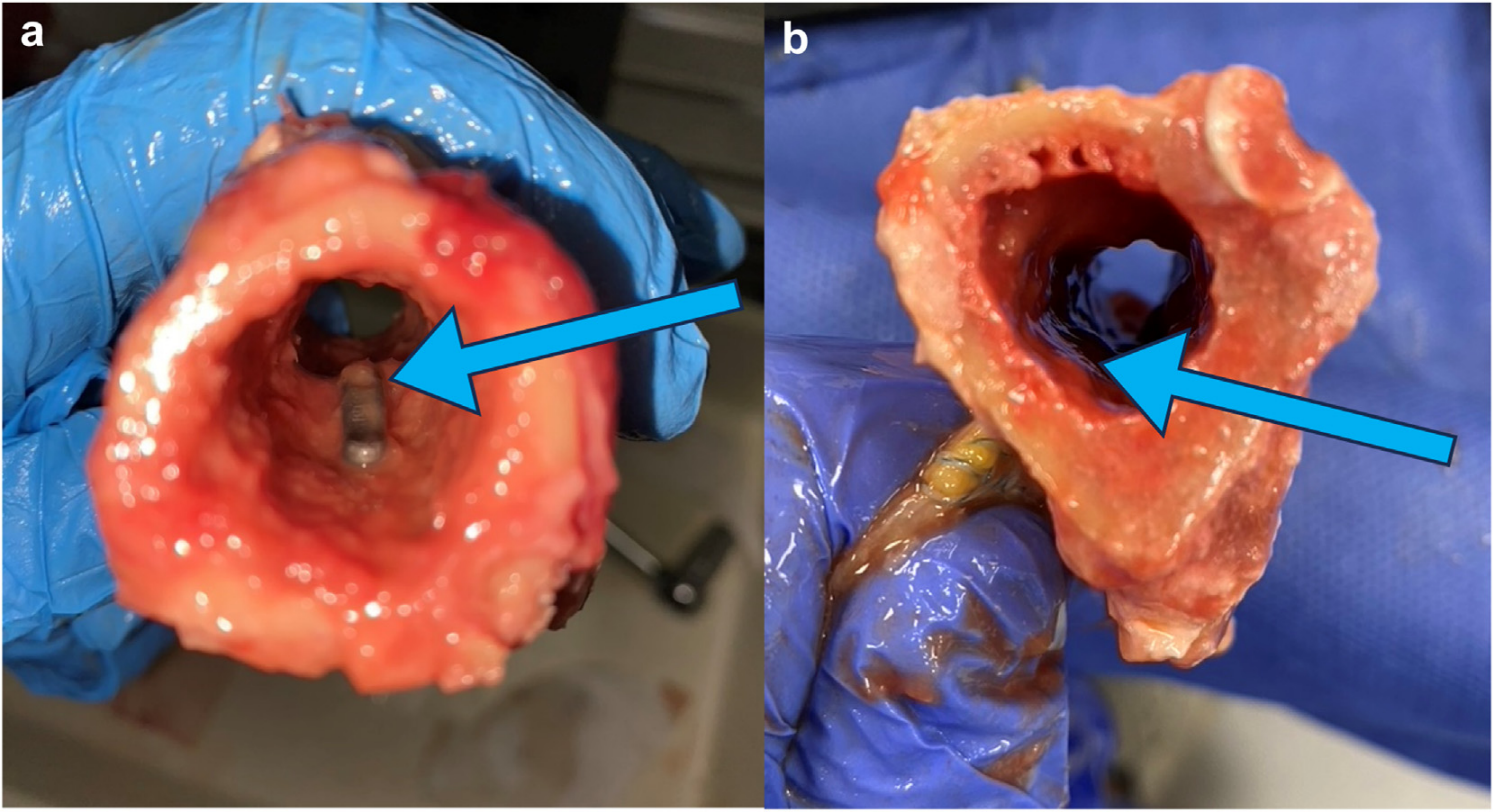
Different set ups: (**a**) unicortical inlay fixation with curved button; (**b**) unicortical onlay fixation with flat button (button is flush against the cortex).

In case of a standard flat unicortical button (ENDOBUTTON; Smith and Nephew, Watford, England, UK), a 3.2-mm hole and in case of newly developed curved button, a 4.5-mm hole was drilled approximately 50 mm distal from the entrance of the bicipital groove, to maintain the right length-tension relationship. The intramedullary button is then inserted into the canal, and the sutures are pulled to flip and secure the cortical button against the inner cortex of the humerus. The sutures were tightened to secure the biceps tendon firmly against the anterior cortex of the proximal humerus in case of a standard flat unicortical button and against the button through the drilled canal in case of the newly developed curved button. The correct position of the button was confirmed by looking through the cross-section of the bone (Fig. 3).

**Figure 4:**
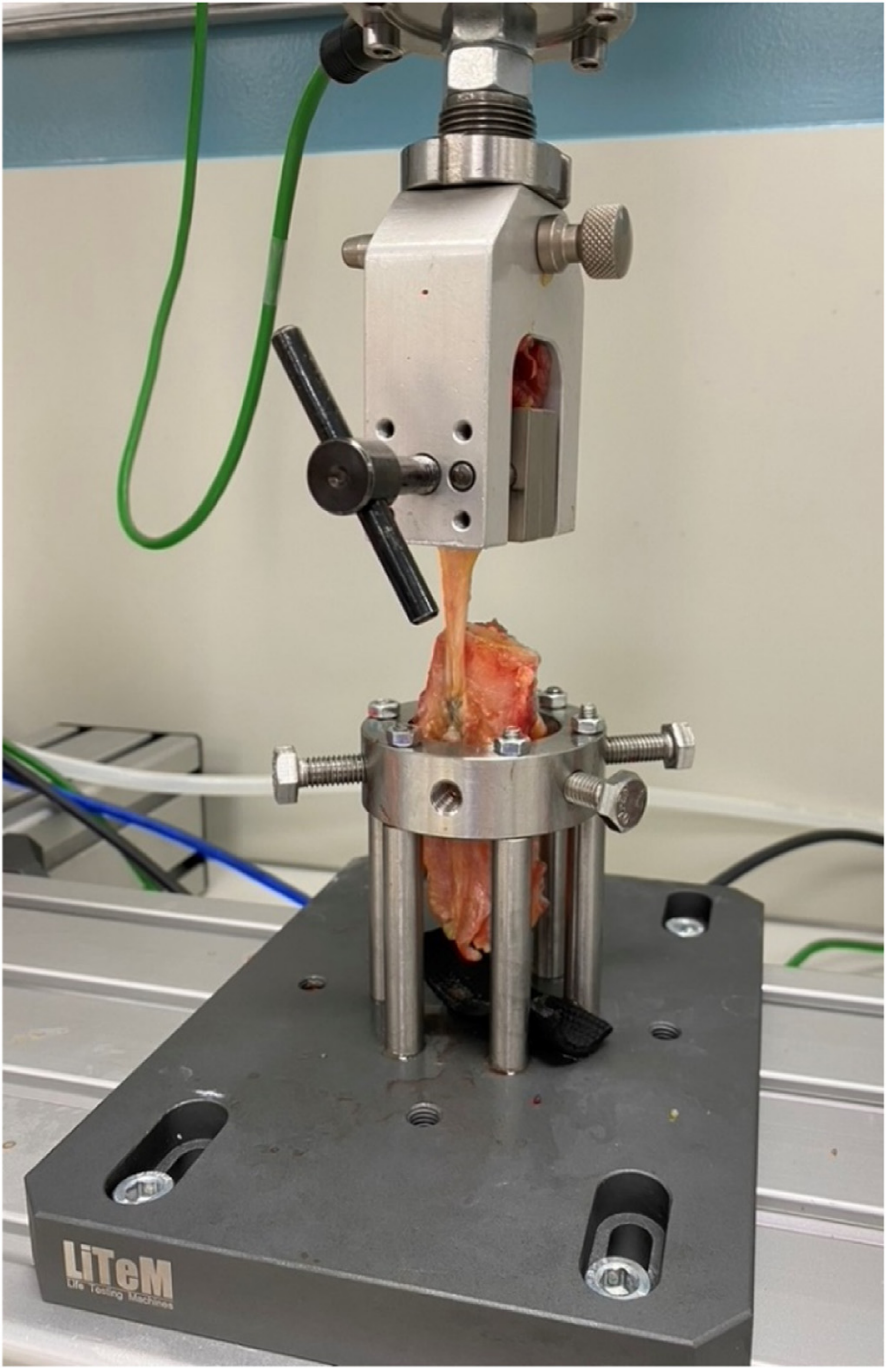
Test set up with custom mount to clamp the humerus to ensure vertical line of pull.

After performing the button placement, all soft tissue was removed except for the long-head biceps tendon, the biceps, and the proximal 10 cm of the humerus. The humerus was clamped inside Litem pneumatic modular frame vertical double-column testing machine with TC-Micro series loas cell (L-TC-M-500) to a custom mount, and the long-head biceps tendon was firmly attached to a metal clamp 2 centimeter distal to the fixation site of the biceps tendon (Fig. 4). The line of pull on the biceps was vertical following the longitudinal axis of the humeral shaft (Fig. 4). This is in accordance with the physiological loading condition.

All specimens were subjected to a cyclic loading test consisting of 1000 cycles at 2.5 Hertz (Hz), ranging from 5 to 100 Newton (N). After the 1000 cycles are completed, the load was returned to 5 N. For displacement measurements, the position of the actuator was recorded before and after cyclic testing. The specimens that did not fail during the cyclic loading protocol were subjected to a continuously increasing load until failure at an extension rate of 10 Newton per second (N/s). The maximum load to failure was recorded during failure of the construct. Data from the load cell were automatically exported to Excel (Microsoft Excel 2018; Microsoft Corp., Redmond, WA, USA). The mode of failure was determined by observing and if necessary, by dissecting the failed construct.

### Statistical analysis

Data were extracted and analyzed with Statistical Package for the Social Sciences (SPSS; IBM Corp., Armonk, NY, USA). Depending on the distribution, an independent samples t-test or a Mann-Whitney U-test was performed to compare the ultimate load to failure, displacement, and stiffness. The normality of the data distribution was assessed with visual inspection of quantile–quantile plots and the Kolmogorove–Smirnov test. A *P* value lower than .05 was considered significant. Stiffness of the construct was calculated using the linear portion of the load-displacement graph from the load-to-failure testing.

## Results

### Specimens

All 16 specimens were included in the data analysis. The group with curved button consisted of six right and two left shoulders. The group with flat button consisted of five right shoulders and three left shoulders.

### Loading tests

No failure occurred during cyclic testing of the constructs. After cyclic testing, the median displacement was 10.4 mm (6.1-16.1) for the group with curved button and 11.9 mm (6.0-43.9) for the group with flat button (*P* = .721). All constructs completed the load-to-failure testing. The biomechanical test results are summarized in Table I. The group equipped with the curved button exhibited an average load to failure of 239.4 ± 35.1 N, while the group using the flat button had an average of 227.0 ± 42.0 N. The difference in ultimate load was not statistically significant (*P* = .534). The mean stiffness was 70.0 ± 10.3 N/mm for the group with the curved button and 61.3 ± 17.3 N/mm for the group with the flat button, which was not statistically significant (*P* = .242). The biomechanical test results are summarized in Table I.

**Table I:** Results of the biomechanical testing.

	Curved button (n = 8)	Flat button (n = 8)	*P* value
Cyclic testing			
Displacement after cyclic loading, mm, median (range)	10.4 (6.1-16.1)	11.9 (6.0-43.9)	.505 (r. = .18)
Load-to-failure testing			
Displacement at failure, mm, median (range)	21.3 (10.7-52.1)	21.9 (16.2-50.5)	.721 (r. = .11)
Ultimate load, N, mean ± SD	239.4 ± 35.1	227.0 ± 42.0	.534 (95% CI = −53.9 to 29.2)
Stiffness, N/mm, mean ± SD	70.0 ± 10.3	61.3 ± 17.3	.242 (95% CI = 23.9-6.6)
Mode of failure, n (%)			-
Suture	3 (37.5)	4 (50.0)	
Tendon	5 (62.5)	4 (50.0)	
Bone	-	-	

*SD*, standard deviation; *CI*, confidence interval.

## Discussion

Mechanical testing replicating the repetitive stress of arm movement through cyclic loading was used to assess the resistance of tenodesis constructs to regular motion and postoperative activity. Load-to-failure testing was applied to measure mechanical strength in harsh circumstances. The results from both cyclic loading and load-to-failure tests suggest that the performance of the newly developed curved button is similar to the performance of the standard flat button.

The biomechanical results of the curved button tenodesis are similar to other currently used tenodesis techniques.^2^^,^^14^ The theoretical advantages of this button are due to the inlay technique enabling tendon placement within the bone tunnel, potentially creating both cortical and intracortical tendon to bone contact. However, this inlay technique requires a larger bone tunnel of 4.5 mm to accommodate both the button and tendon. In comparison, some onlay techniques utilize smaller implants, allowing for a smaller drill hole of just 2.6 mm. Although larger tunnels decrease the tortional strength of the humeral bone, the biomechanical strength of the 2.6 mm vs. 4.5 mm tunnel for proximal biceps tenodesis remains untested.^15^ A biomechanical study comparing the torsional strength of the humerus with either a 6.25 mm or 8.0 mm tunnel revealed no significant differences between the two tunnel sizes.^3^ Therefore, while concerns about fracture risk and tunnel size are valid and supported by general orthopedic principles, it remains debatable whether the difference between 2.6 mm and 4.5 mm tunnel would translate into clinically significant differences in fracture rates; especially, considering that the incidence if humeral fractures in proximal biceps tenodesis patients is reported to be less than 0.1%.^11^

To date, there is no clear consensus on whether healing within the bone tunnel (inlay) or cortical surface (onlay) provides superior outcomes. In a rabbit model, Tan et al found no significant difference between groups in terms of failure load, stiffness, and mean volume of newly formed bone when comparing tendon-to-bone healing.^18^ The histological analysis showed minimal tendon-to-bone binding inside the bone tunnel and similar healing on the outer surface for both onlay and inlay techniques. In contrast, study by Gao et al on a rat model found that cancellous (inlay) fixation resulted in higher failure load, stiffness, and proportion of new bone 4 weeks postoperation, even though no difference in failures was observed.^10^ However, 8 weeks postoperation, there were no significant differences in biomechanics, new bone formation, and histology between the two groups.^10^ Therefore, cancellous fixation might provide stronger fixation during the initial weeks postsurgery, potentially resulting in a lower tenodesis failure rate during early rehabilitation programs and allowing more aggressive rehabilitation. Current study showed that the fixation with the curved button is as strong as with a flat button. Considering these findings, this button design could be used in in-vivo testing to test the theoretical advantages of this curved button.

**Figure 5:**
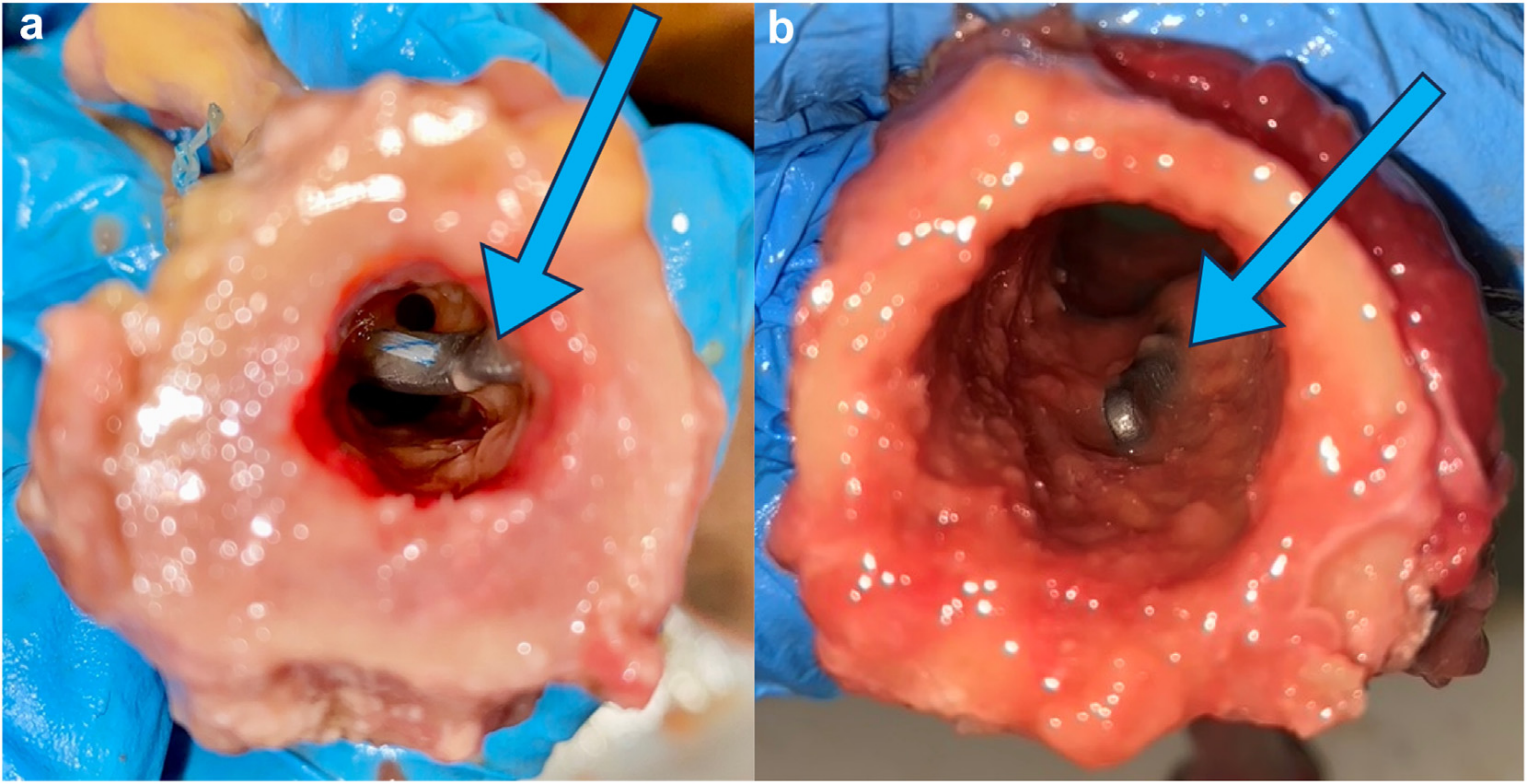
Different concerns: (**a**) curved button catching against the posterior cortex; (**b**) curved button flipped on its side.

During the course of this study some potential challenges were observed in relation to the application of the curved button. First, the new curved button is larger in length, which is necessary to bridge the length of radial tuberosity for distal biceps tenodesis. However, when placing this button during proximal biceps tenodesis, it can get caught against the posterior cortex when inserting it in the medulla (Fig. 5). Consequently, the placement of the button may be more challenging, given that the length of the button exceeds the diameter of the humeral medullary cavity, which is approximately 15 mm at the location of subpectoral tenodesis.^16^ This is less likely to occur during suprapectoral or bicipital groove tenodesis considering the humerus diameter is bigger in more proximal locations. Interestingly, this was not observed in another cadaveric study that tested this curved button for distal biceps tenodesis.^5^ Within that study, a larger tunnel with the diameter of 8 mm was used due to a larger thickness of the distal biceps tendon. Therefore, the button could be inserted into the tunnel at a less perpendicular angle, which likely made the placement easier. A second potential concern is the positioning of the button inside the humerus. When tightening the button with the suture, it may flip on its side (Fig. 5). As a result, the tendon will be pulled in only at depth of the humeral cortex, which is on average 4.4 mm.^19^ When the button is flipped on its side, it is flush with the cortex like the standard flat button. Therefore, to prevent the button from flipping, a knot pusher could be used while inserting the button. When inserting the button, one wire thread should run through the pusher and another alongside it. Once the button is in the hole, tension is applied to the wire running through the pusher, causing the button to stand upright. Maintaining this tension prevents the button from flipping. Fluoroscopy may be needed to check the positioning of the button during the operation. Moreover, the inlay technique requires a larger drilling hole to secure the tendon inside the tunnel. This could lead to a higher fracture risk, as the bone strength is correlated with defect size.^9^ However, humeral fracture after proximal biceps tenodesis is rare for all fixation methods, as it occurs in 0.5% of biceps tenodesis patients.^11^

### Limitations

This study should be interpreted with the following limitations in mind. Firstly, this was a time-zero, in-vitro biomechanical study. Potential healing advantages of the unicortical inlay technique were not assessed. Secondly, there was no information available regarding the human cadaveric specimens that were used within this study. These specimens were donated for the purpose of medical research, and unfortunately within the department where these specimens were prepared, no records were accessible after separation of limbs. However, based on the average life expectancy in the Netherlands (70-90 years), it can be assumed that the age of the specimens used in this study is likely comparable to those used in similar research. This assumption is further supported by the fact that our findings align with those from other studies. Thirdly, our study utilized a relatively small sample size. Despite this, it is important to highlight that the number of specimens used is consistent with other published biomechanical studies. Moreover, our displacement measurements were derived from actuator position, rather than from image-based tracking of the tendon and bone. This means, we were unable to determine the precise source of displacement within the construct.

## Conclusion

In this time-zero in-vitro study, the curved and flat buttons exhibited similar biomechanical properties in terms of displacement, load to failure, and stiffness. Considering these results and the theoretical advantages of the curved button, this technique could be a new alternative for the treatment of proximal long-head biceps tendon pathology.

## Acknowledgment

The authors would like to thank A.Bruseker and A.Shirinskaya for creating the medical illustrations and designs of Figure 1.

## Disclaimers

Funding: This research was supported by funding from Arthroscopy & Arthroplasty Courses Utrecht 2023 and Orthopaedic Research Foundation Genk. The funding was specifically used to cover the costs of renting a tensile testing machine, with the funds being directly transferred to the company providing the equipment.

Conflicts of interest: The authors, their immediate families, and any research foundation with which they are affiliated have not received any financial payments or other benefits from any commercial entity related to the subject of this article.
